# Visualizing the IKEA effect: experiential consumption assessed with fNIRS-based neuroimaging

**DOI:** 10.3389/fnrgo.2023.1129582

**Published:** 2023-04-17

**Authors:** Hiroki Oishi, Kenta Nakazawa, Tomoki Takahashi, Yasushi Kyutoku, Ippeita Dan

**Affiliations:** Applied Cognitive Neuroscience Laboratory, Faculty of Science and Engineering, Chuo University, Bunkyo, Japan

**Keywords:** experiential consumption, IKEA effect, functional near-infrared spectroscopy (f-NIRS), personalized adaptive GLM, DIY—do it yourself, memory retrieval, attachment

## Abstract

**Introduction:**

In recent years, experiential consumption, which refers to purchases involving hedonic experiences, has been gathering attention in marketing research. Experiential consumption is closely related to cognitive biases, and among them, we focus on the IKEA effect, which is a cognitive bias in which the maximum willingness to pay (WTP) for a product is high because the experience of assembling the product is highly valued. Since no studies have examined the neural mechanism behind the IKEA effect, here we present the first study exploring the neural substrates of the IKEA effect using functional near-infrared spectroscopy (fNIRS). During the WTP evaluation, we expect the attachment to and memory retrieval of DIY products to be the cognitive mechanism for the IKEA effect.

**Methods:**

Thirty healthy students, of which 24 were confirmed to have undergone the IKEA effect, were asked to perform a WTP evaluation task after assembling three types of do-it-yourself (DIY) products and handling three types of Non-DIY products. Their cerebral hemodynamic responses during the evaluation were measured using fNIRS. In order to adjust for temporal variability of cortical responses among participants, a personalized adaptive general linear model (GLM) analysis was adopted. Then, one-sample *t*-tests were performed for each DIY and Non-DIY condition for the obtained β values, and a paired *t*-test was performed between DIY and Non-DIY conditions.

**Results:**

We identified brain regions, including the left-inferior frontal gyrus (L-IFG) and left-middle frontal gyrus (L-MFG), which were probably related to cognitive processing related to the IKEA effect. Among them, the L-MFG exhibited more activation during the DIY condition than during the Non-DIY condition.

**Conclusion:**

To our knowledge, the current study is the first to reveal the neural basis of the IKEA effect. The cortical activation during evaluation of WTP for DIY and Non-DIY products exhibited marked differences. In addition to the R-IFG activation often reported for WTP evaluations, we revealed that other regions, in particular the L-IFG and L-MFG, were activated during the DIY condition. These areas are considered to be related to memory and attachment, which would serve as reasonable cognitive constituents for the IKEA effect. In conclusion, this study suggests that the value of experiential consumption can be assessed using fNIRS-based neuroimaging and provides a novel approach to consumer neuroergonomics. It is predicted that visualization the value of experiential consumption will create marketing opportunities for more and more companies and the visualization will become an indispensable method in the future.

## Introduction

In recent years, experiential consumption, which refers to purchases of hedonic experiences, has been gathering attention in marketing research (Wang et al., 2020). Experiential consumption as identified by Holbrook and Hirschman (1982) is different from utilitarian consumption and it is perceived as hedonistic consumption pursuing fun, fantasies, and feelings. Many studies have shown that the experiential consumption of products generates a higher level of happiness than material consumption (Van Boven and Gilovich, 2004; Bigné et al., 2008; Carter and Gilovich, 2010; Kumar et al., 2014; Zhang et al., 2014; Gilovich et al., 2015; Walker et al., 2016). In addition, Li and Lee (2016) conducted a study in a resort hotel and found that customer satisfaction was positively related to the perceived experiential consumption framework developed by Schmitt (1999), which consists of five elements (feel, sense, think, relate, and act). Thus, experiential consumption is becoming a major trend as a marketing strategy to attract consumers as well as a new genre of market in its own right.

Interestingly, another type of marketing strategy called “sensory marketing,” which is similar to experiential consumption, is also gaining attention (Bhatia et al., 2021). Sensory marketing refers to marketing that focuses on consumers' five senses and influences their perceptions, decisions, and behaviors (Krishna and Morrin, 2007). However, such processes are substantially affected by cognitive biases. For example, when a consumer is purchasing something, the difference between what they expect based on visual information and the actual content of the product significantly affects their evaluation. Satisfaction may vary due, for example, to the difference between the amount of a product that the consumer judges will be contained based on the package shape and the actual amount contained (Raghubir and Krishna, 1999). In addition, there are other effects, such as tactile information obtained at the last minute, which may affect the consumer's judgment and evaluation. For example, changing the material of the container from which a drink is consumed can change the consumer's evaluation of the drink (Krishna and Morrin, 2007).

Recently, new types of cognitive biases that involve a series of product-related events and thus entail multiple cognitive biases in a composite manner, have also been proposed. One of these is the IKEA effect, which has been found to occur when the experience of creating or assembling an object leads to a higher appreciation of the product's value. Specifically, the IKEA effect refers to a cognitive bias in which the willingness to pay (WTP) is high because assembling discrete products by oneself is highly valued (Norton et al., 2012) and is closely related to experiential consumption. WTP is the maximum price that a consumer is willing to pay for a certain product. A study by Norton et al. (2012), proved the IKEA effect by comparing WTP score with and without assembling IKEA products and folding origami. The experiment revealed three characteristics that lead to the IKEA effect. First, the IKEA effect is caused by assembling IKEA products and origami products. Second, merely disassembling a product does not cause the IKEA effect. Third, the IKEA effect occurs only once the product assembly is complete. Norton et al. (2012) indicated that the IKEA effect is a compound effect which increases WTP due to attachment and memory of assembling experiences to a DIY product resulting from the effort put into it. It is also possible that the experience of assembling products during the IKEA effect could activate areas related to memory retrieval. Similarly, Atakan (2011) study also describes the attachment that occurs through DIY activities. However, the cognitive mechanisms behind the IKEA effect, particularly its neural substrate, have not yet been clarified. Therefore, we aimed to explore the cognitive mechanisms caused by the IKEA effect from the perspective of brain functions.

In clarifying the cognitive mechanism of the IKEA effect, we focused on brain functions during WTP evaluations and brain functions related to attachment and memory retrieval. Plassmann et al. (2007) used functional magnetic resonance imaging (fMRI) to examine brain activation during WTP evaluation and reported that the right medial orbitofrontal cortex (R-mOFC) and the right dorsolateral prefrontal cortex (R-DLPFC) were activated. They also confirmed a positive correlation between the R-DLPFC and WTP. Votinov et al. (2010) examined brain activity for endowment effects along with WTP evaluation using fMRI and found that six regions, including the bilateral anterior cingulate cortex, and bilateral medial frontal cortex (bilateral-MFC), were activated during WTP evaluation. The monetary amount and the cortical activation exhibited a positive correlation in the right inferior frontal gyrus (R-IFG). Moreover, studies by Kawabata Duncan et al. (2019) and Hirabayashi et al. (2021) used fNIRS to examine brain activation during WTP evaluation for cosmetic products and found a positive correlation between the R-DLPFC activation during foundation and lipstick application and WTP score for each participant. Among these regions, the R-IFG plays a role in attentional control and economic decision-making (Dehaene et al., 1998; Duncan, 2001), and when evaluating WTP, attentional control and decision-making are said to occur. On the other hand, the bilateral-MFC has been said to be activated when considering the WTP evaluation of a product, as it has been found to be significantly correlated with loss avoidance behaviors (Tom et al., 2007) and conflict of wills (Nachev et al., 2005). In addition, the R-DLPFC is a brain area that is activated when evaluating non-verbal information (Kelley et al., 1998; Opitz et al., 2000; Rothmayr et al., 2007; Blanchet et al., 2010; Okamoto et al., 2011; Savini et al., 2012) and is activated when judging the value of products (Watanabe, 1996; Wallis and Miller, 2003). Therefore, R-DLPFC activation during WTP evaluation is considered relevant. In summary, based on these previous studies, we expected that the R-IFG, and R-DLPFC would be activated during WTP evaluation.

Next, we will consider cortical activations related to attachment, an important cognitive component of the IKEA effect that is expected to develop during a consumer's assembly of a product. Two types of attachment have been revealed in prior research: attachment to products and to people. Additionally, many studies have indicated that the underlying mechanisms of attachment are common, even if the target is different (Belk, 1988; Wallendorf and Arnould, 1988; Schultz et al., 1989; Dwayne Ball and Tasaki, 1992; Kleine III et al., 1993). For this reason, we refer to two types of previous studies. Kikuchi et al. (2021) used fMRI to measure brain regions associated with attachment to cosmetics and reported that the left ventral pallidum (L-VP) and right posterior cingulate cortex (R-PCC) were activated. Minagawa-Kawai et al. (2008) used fNIRS to measure brain functions related to attachment between mothers and infants and reported that the anterior orbitofrontal cortex (OFC) was activated. Note that the anterior OFC is contiguous to the frontopolar cortex (FPC), and signal separation between the two regions using fNIRS is difficult. Quevedo et al. (2017) used fMRI to examine brain regions associated with attachment and found that three regions, including the L-DLPFC, are associated with attachment. The ventral PCC is said to be the core region of the self-referential processing system (Smith et al., 2009). The OFC is said to be an essential brain region for emotional regulation (Blair et al., 1999). Considering these studies, if attachment to people and attachment to products both occur through similar mechanisms, we can predict that the anterior OFC (or FPC), and L-DLPFC, among the regions to be examined by fNIRS, will be activated due to the IKEA effect, which Norton et al. (2012) suggested was associated with attachment.

Finally, we discuss the brain regions related to memory, an essential cognitive component of the IKEA effect that is expected to require the memory retrieval during the WTP evaluation task. Ranganath et al. (2004) found that the FPC is involved in associative memory retrieval using fMRI. Several studies have reported that the FPC is associated with memory recall (Lepage et al., 2000; Cabeza et al., 2003), and it is said to be related to memory retrieval. Miyashita (2004) in their fMRI study on memory recall, revealed that regions such as bilateral-IFG and L-MFG (a major part of DLPFC) were activated. Therefore, activation of the FPC, bilateral IFG, and L-DLPFC can be predicted in the present study as well.

Since the IKEA effect has been shown to occur in everyday situations, we must measure brain activation during the IKEA effect in everyday situations. fNIRS is non-invasive, not harmful to the body (Strangman et al., 2002; Ferrari and Quaresima, 2012), and has been reported less sensitive to motion artifacts (Scholkmann et al., 2010; Huang et al., 2022). Owing to these characteristics, fNIRS is particularly well suited for research in the context of everyday life (He et al., 2021), making it possible to measure brain function with high accuracy even in everyday environments. In addition, a variety of studies have been conducted using fNIRS to measure brain activity related to consumer behavior. For example, fNIRS has been used for WTP evaluation of cosmetics (Kawabata Duncan et al., 2019; Hirabayashi et al., 2021) and for consumers' decision-making behavior when envisioning shopping (Krampe et al., 2018). For these reasons, we determined that the use of fNIRS was appropriate for studying the neural substrate of the IKEA effect in an everyday situation.

The purpose of this study was to confirm that the IKEA effect occurs in the present experimental design and to examine the cognitive mechanisms behind the IKEA effect from the perspective of brain function. Specifically, we hypothesized based on previous research that WTP score for a DIY condition is higher than for a Non-DIY condition. For brain functions, we expected that either the FPC, bilateral DLPFC (including MFG), and/or bilateral-IFG could be activated in association with the evaluation of WTP and the attachment and memory retrieval thought to be developed through the DIY-style of product assembly by the consumer.

## Method and materials

### Participants

This experiment began with thirty participants (all students, mean age 21.17 ± 1.93 years, range 18–26, 15 males and 15 females). All participants were right-handed, and native speakers of Japanese. Four participants for whom, based on their mean WTP scores, the IKEA effect did not occur (WTP for the three DIY products was not higher than or the same as the mean WTP for the three Non-DIY products), and two participants who reported typing errors were excluded from subsequent analyses. As a result, the data for 24 participants were analyzed. The remaining 24 participants confirmed in the post-experiment questionnaire that they had never purchased or assembled any of the six products used in this experiment. In addition, 23 participants had never seen any of the six products. One participant indicated that he had seen the six products, but not assembled them. However, because there was a discrepancy between the actual price and the market price predicted in the post-experiment questionnaire, it was determined that this did not affect his WTP response. Before the experiment, informed consent was obtained from all participants. Participation was voluntary, and an honorarium as well as the six IKEA products used for the experiments were given to the participants after the experiment. This study was conducted after obtaining permission based on the Declaration of Helsinki from the Ethics Committee at Chuo University.

### Procedure

Participants were informed in advance that they should not eat or ingest caffeine for 2 h prior to the experiment. Just before the fNIRS measurements, participants responded to two questionnaires. We conducted Edinburgh's dominant hand test and confirmed that participants were right-handed (Oldfield, 1971). The Mini International Neuropsychiatric Interview (M.I.N.I.) was used to screen out participants with psychiatric disorders (Sheehan et al., 1998) because Blumberg et al. (2003) and Aupperle et al. (2012) reported that brain activation in psychiatric patients with PTSD and bipolar disorder is more filtered than that in healthy individuals. After the study was completed, we told the participants that they would be given these six products.

In order to determine which six products would be appropriate for the current study, we conducted a preliminary questionnaire survey in June 2020 that targeted 13 out of over 1,500 IKEA products available at IKEA stores in Japan and priced at 999 yen. The preliminary questionnaire included WTP, free-entry survey of projected market prices, and product evaluation using the five-item method. Three DIY products with similar difficulty levels were selected based on the questionnaire results and their respective assembly instructions, and three Non-DIY products were selected based on the questionnaire results. The results of the preliminary questionnaire confirmed that there was no significant difference in WTP scores between the six IKEA products [*F*_(5,135)_ = 0.727, *p* = 0.61].

### Experimental design

**Figure 1:**
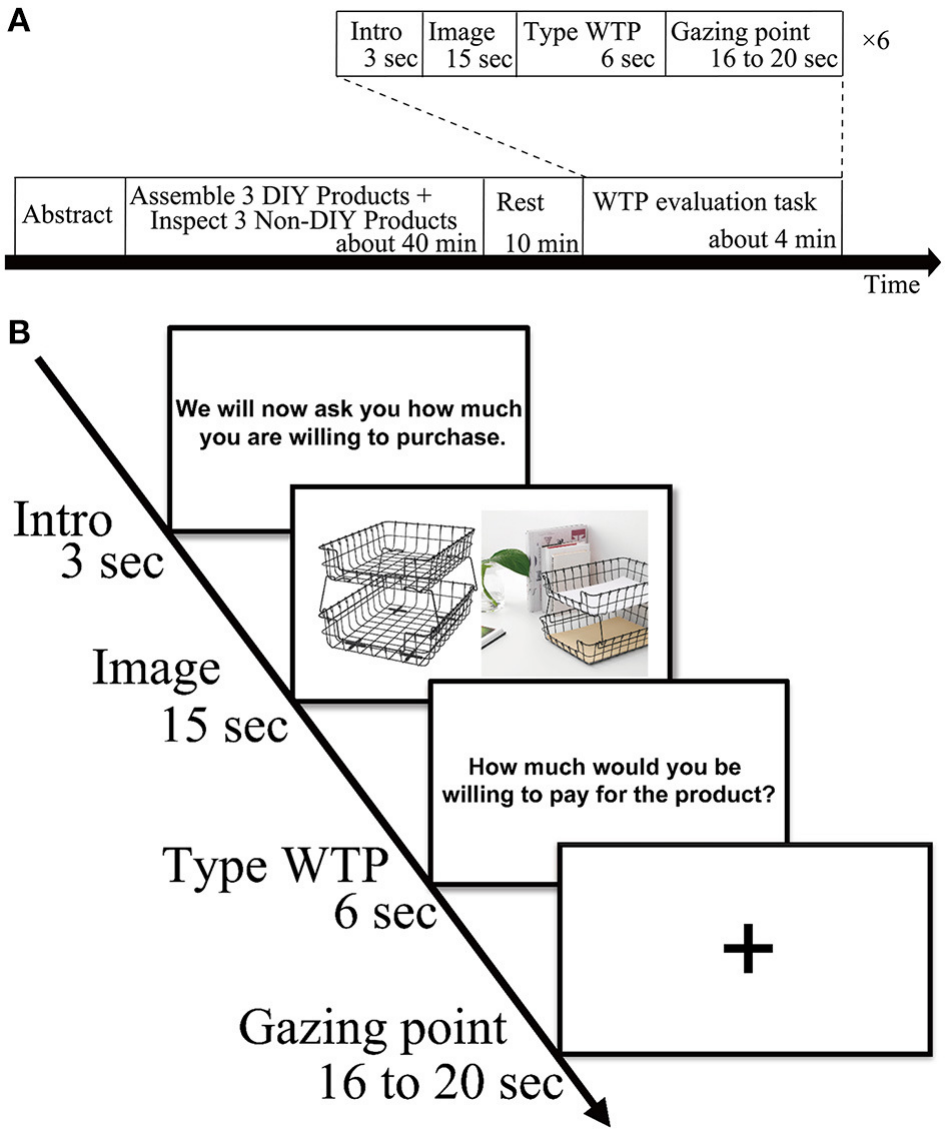
**(A)** Schematic showing an experimental block. The order of assembling and handling was random. **(B)** Trial procedure. The order of product images was randomized.

Experimental procedures are depicted in Figure 1. Before the fNIRS measurement, participants were asked to assemble the three DIY products and to inspect the three Non-DIY products for at least 1 min. We randomized and alternated the order of the DIY condition and the Non-DIY condition to eliminate order effect. Participants were then given a 10-min break followed by the WTP evaluation of the six products, which was taken along with the fNIRS measurements. The entire experiment lasted about an hour. The WTP evaluation task was conducted for each of the six products (three products in the DIY condition and three products in the Non-DIY condition) for each participant in an event-related design. The order of the products in the WTP evaluation task was randomized among the participants. For the WTP evaluation task, an experimental program was created using E-prime 2.0 (Psychology Software Tools, Pittsburgh, PA, USA). All the directions displayed during the task were written in Japanese. During the experiment, the participants were asked to follow the directions displayed on the monitor. For instructional purposes, the screen displayed “Later we will ask you how much you would be willing to pay for this item.” for 3 s and then the images of the six products were randomly displayed for 15 s each. Then, “How much would you be willing to pay for the product?” was displayed for 6 s and the participants entered their WTP amount. In addition, a gazing point was shown on the screen for 16–20 s at random. This process was repeated six times to evaluate each participant's WTP for the three Non-DIY products and the three DIY products. The WTP evaluation task took about 42 s per trial, so it took about 4.2 min total to measure the six products. Before the measurements were taken, the WTP evaluation task was practiced on three products unrelated to the experiment.

### fNIRS data acquisition

**Figure 2:**
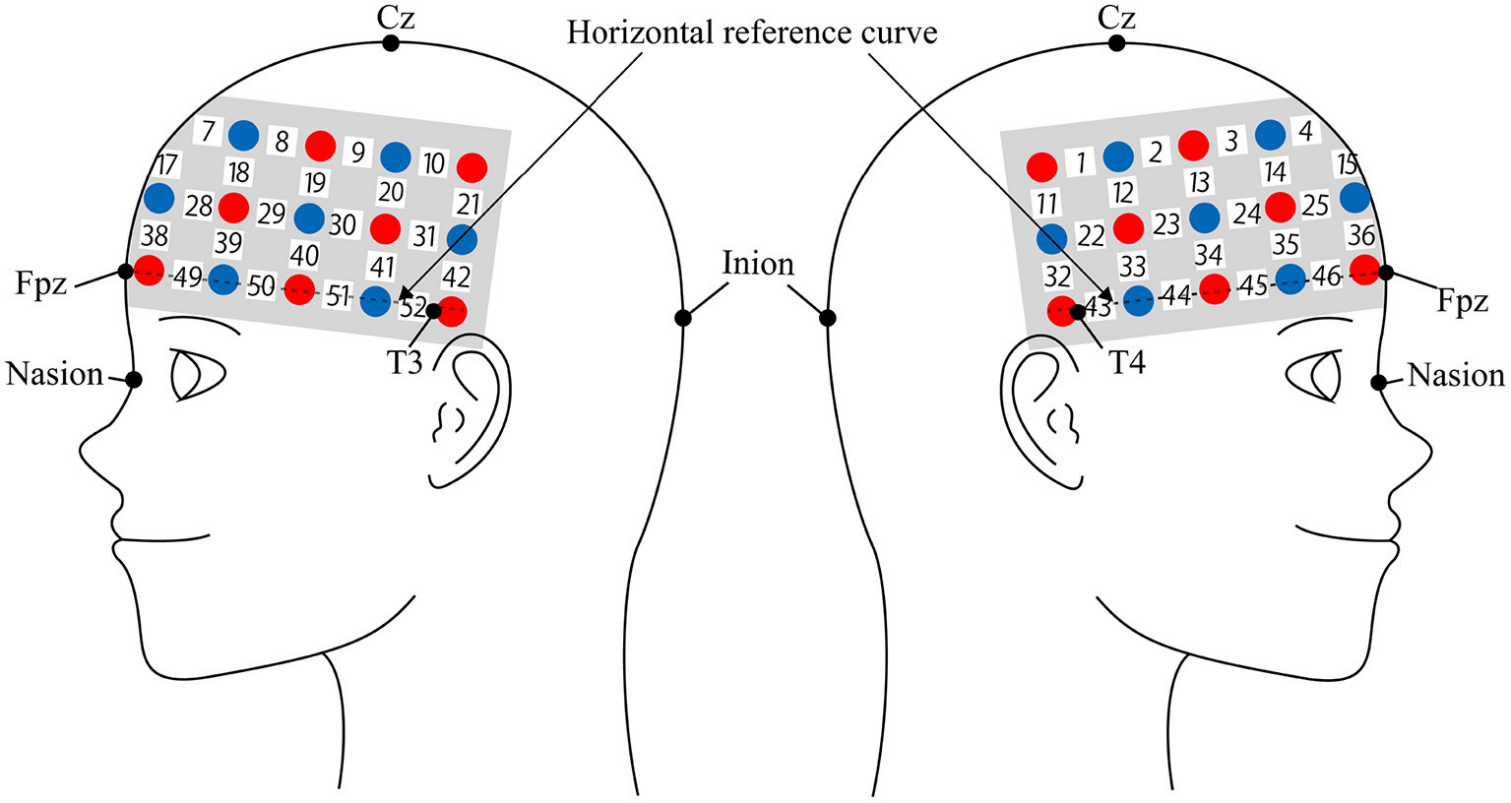
Spatial profiles of functional near infrared spectroscopy (fNIRS) channels. Left and right side views of the probe arrangements are shown with fNIRS channel orientation. Detectors are indicated with blue circles, illuminators with red circles, and channels with white squares. Corresponding channel numbers are shown in black.

We used the Hitachi ETG-4000 to measure cerebral blood flow response using two near-infrared light sources (695 and 830 nm). We analyzed the fNIRS data using a modified Beer-Lambert law (Cope et al., 1988). This method is used to calculate the change of oxyhemoglobin (oxy-Hb) and deoxygenated hemoglobin (deoxy-Hb) signals in units of millimolar × millimeter (mM × mm) based on the intensity change of near-infrared light (Maki et al., 1995). The sampling rate was set at 10 Hz. In this experiment, we used 3 × 11 multichannel holders (Figure 2). The probe used in the experiment consisted of 17 illuminator and 16 detector alternately spaced 3 cm apart, for a total of 52-channel sites measured. The probe was mounted according to the international 10–20 system as a reference point. First, the multichannel probe holder was placed such that the detector in the middle of the lowest row corresponded to Fpz. Then, the illuminators and detectors in the lowest row were matched to the horizontal reference curve, which was determined by a straight line connecting T3-Fpz-T4 (Klem, 1999; Jurcak et al., 2007).

### Registration of fNIRS channels to MNI space

After measuring cerebral blood flow, the location of all the optodes and the landmarks such as Nasion, Inion, CZ, and bilateral Preauricular Reference Points were acquired using the Polhemus Patriot digitizer (Polhemus, Colchester, VT, USA). We employed the probabilistic registration to register fNIRS data to Montreal Neurological Institute (MNI) standard brain space (Tsuzuki et al., 2007; Tsuzuki and Dan, 2014). The projected spatial data were mapped to anatomical labels (Okamoto et al., 2004; Okamoto and Dan, 2005). The spatial registration data were registered with macro-anatomical labeling (Okamoto et al., 2004; Okamoto and Dan, 2005) in reference primarily to Brodmann's atlas (BA) (Rorden and Brett, 2000) and secondarily to the macro-anatomical labeling in LPBA40 (Shattuck et al., 2008).

### Preparation for GLM analysis

In the first-level analysis, time series data for oxy-Hb and deoxy-Hb were analyzed using in-house MATLAB analysis tools developed in the Applied Cognitive Neuroscience Laboratory at Chuo University (available upon request). Oxy-Hb was primarily used in the analysis, because it is said to have a higher hemodynamic response than other signals (Huppert et al., 2006). First, each channel was pre-processed for oxy-Hb time series data. Channels with signal fluctuations of 10% or less were considered to have poor measurement quality and were excluded from the analysis. After exclusion, wavelet minimum description length (Wavelet-MDL) was applied to the remaining channels (Jang et al., 2009). Wavelet-MDL is a method for removing global trends due to respiration, heartbeat, and vascular motion, and does not require the definition of Hz for optional drifts.

Basis functions used for GLM analysis were generated from the hemodynamic response functions (HRFs) *h*(τ_*p*_, *t*) which were created as in Equation 1 (Friston et al., 1998).


(1) $$\begin{array}{l} \text{h}\left({\text{τ}}_{\text{p}},\text{t}\right)=\frac{{\text{t}}^{\tau_{\text{p}}}e^{-\text{t}}}{\left(\tau_{\text{p}}\right)!}-\frac{{\text{t}}^{\tau_{\text{p}}+\tau_{\text{d}}}{\text{e}}^{-\text{t}}}{A\left(\tau_{\text{p}}+\tau_{\text{d}}\right)!}, \end{array}$$

where t represents a point in the time series, τ_p_ represents the first peak delay, and τ_d_ represents the second peak delay. A is the amplitude ratio between the first and second peaks and was set to 6 as in typical fMRI studies. τ_p_ was adjusted as described below according to the adaptive GLM (Uga et al., 2014), and τ_d_ was set to 10 s after τ_p_ as in typical fMRI studies. Basis functions *f*(τ_*p*_, *t*) were generated by convolving the HRF *h*(τ_*p*_, *t*) with a boxcar function *u*(*t*),


(2) $$\begin{array}{l} \text{f}\left({\text{τ}}_{\text{p}},\text{t}\right)=h\left(\tau_{\text{p}},\text{t}\right)⊗u\left(\text{t}\right), \end{array}$$

where the symbol ⊗ denotes convolution integral. The basis functions were used to compose each regressor as described below.

For each channel, GLM analysis with regression to the basis functions was performed after preprocessing. The GLM analysis is generally presented in Equation 3,


(3) $$\begin{array}{l} \text{Y}=\text{Xβ}+\text{ε},\text{β}={\left[{\text{β}}_{1},\;\beta_{2},\;\cdots \;,\beta_{N}\right]}^{\text{t}}, \end{array}$$

where Y is a vector with M elements representing the hemodynamic response signal intensity corresponding to the oxy-Hb parameters. *X* is an *M*×*N* design matrix with N being the number of regressors. β is a coefficients vector with N elements. In the current study, β_1_ corresponds to the regressor for product image presentation, β_2_, for typing WTP, and β_3_ represents a constant.

Finally, the least-squares estimation of β is given in Equation 4.


(4) $$\begin{array}{l} \beta ={\left({\text{X}}^{\text{T}}X\right)}^{-1}{\text{X}}^{\text{T}}\text{Y}, \end{array}$$

where X^T^ denotes the transpose matrix of X.

To apply the personalized adaptive GLM, which will be described later, *t*-values (*t*_value_) were obtained to determine the optimized τ_p_ for each participant so that *t*_value_ reached maximum. *t*_value_ was calculated from the regression coefficient β and the residual ε using Equation 5,


(5) $$\begin{array}{l} {\text{t}}_{\text{value}}=\frac{{\text{c}}^{\text{T}}\beta }{\sqrt{\varepsilon^{2}{\text{c}}^{\text{T}}\text{c}{\left(\text{XX}\right)}^{-1}c}}, \end{array}$$

where c is the contrast vector ([1, 0, 0] in this case) and determines the components of the regression coefficient β. Note that the thus-obtained *t*_value_ was not used for null hypothesis testing but as an indicator for τ_p_ optimization.

### Personalized adaptive GLM analysis

The HRF used to model changes in the blood oxygenation level dependent (BOLD) signal in fMRI studies is a regression function that emulates the actual hemodynamic response being measured. Nowadays, HRF is also often used in fNIRS. Boynton et al. (1996) showed that a gamma function with two free parameters, τ_p_ and τ_d_, adequately represents hemodynamic responses.

Uga et al. (2014) reported an adaptive GLM that yields the most effective HRF by selecting the optimal τ_p_. It has been reported that the WTP evaluation time: reaction times (RTs) were influenced by the WTP from individual to individual (Dezwaef et al., 2020). It can be predicted that different RTs will affect τ_p_. Thus, we compared the *R*^2^ values for conventional and personalized adaptive GLM in this study and found that the overall median *R*^2^ was 0.323 for GLM with a fixed τ_p_ of 6, 0.800 for the personalized adaptive GLM of the DIY condition and 0.821 for a personalized adaptive GLM of the Non-DIY condition. Thus, in this study, we optimized HRF to each individual (personalized adaptive GLM), the details of which are to be described in “Preparation for GLM analysis” section as we mentioned above. This method identifies HRF to represent brain functions by selecting the optimal τ_p_ for each individual.

**Figure 3:**
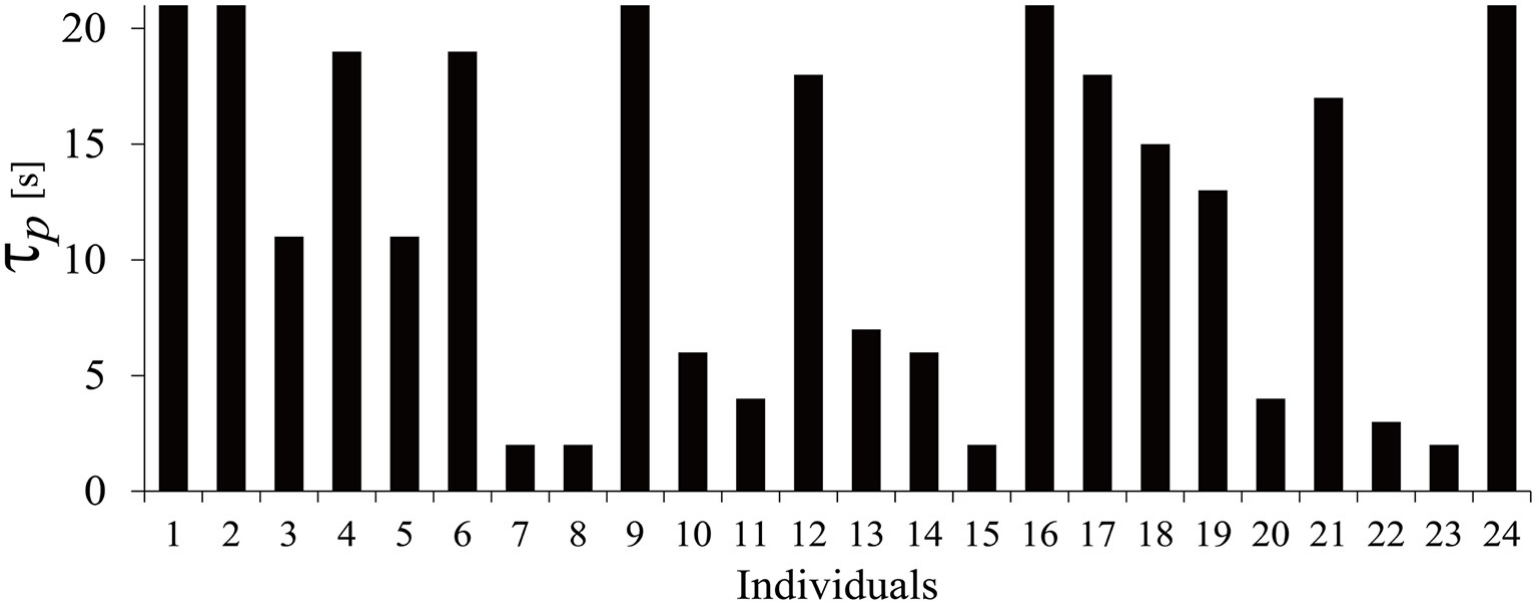
Distribution of τ_p_.

In this study, we have six regressors corresponding to the six products. The optimum τ_p_ for each participant was calculated for the hemodynamic response data of those six products. The value of τ_p_ varied between individuals from 2 to 21 s, which includes product image display and WTP typing. To reduce complication, the second peak delay τ_d_ and amplitude ratio A were set to the typical values (τ_d_ = 10 s, A = 6). The appropriate τ_p_ was specified based on *t*-values. The resulting τ_p_ for each individual is shown in Figure 3.

**Figure 4:**
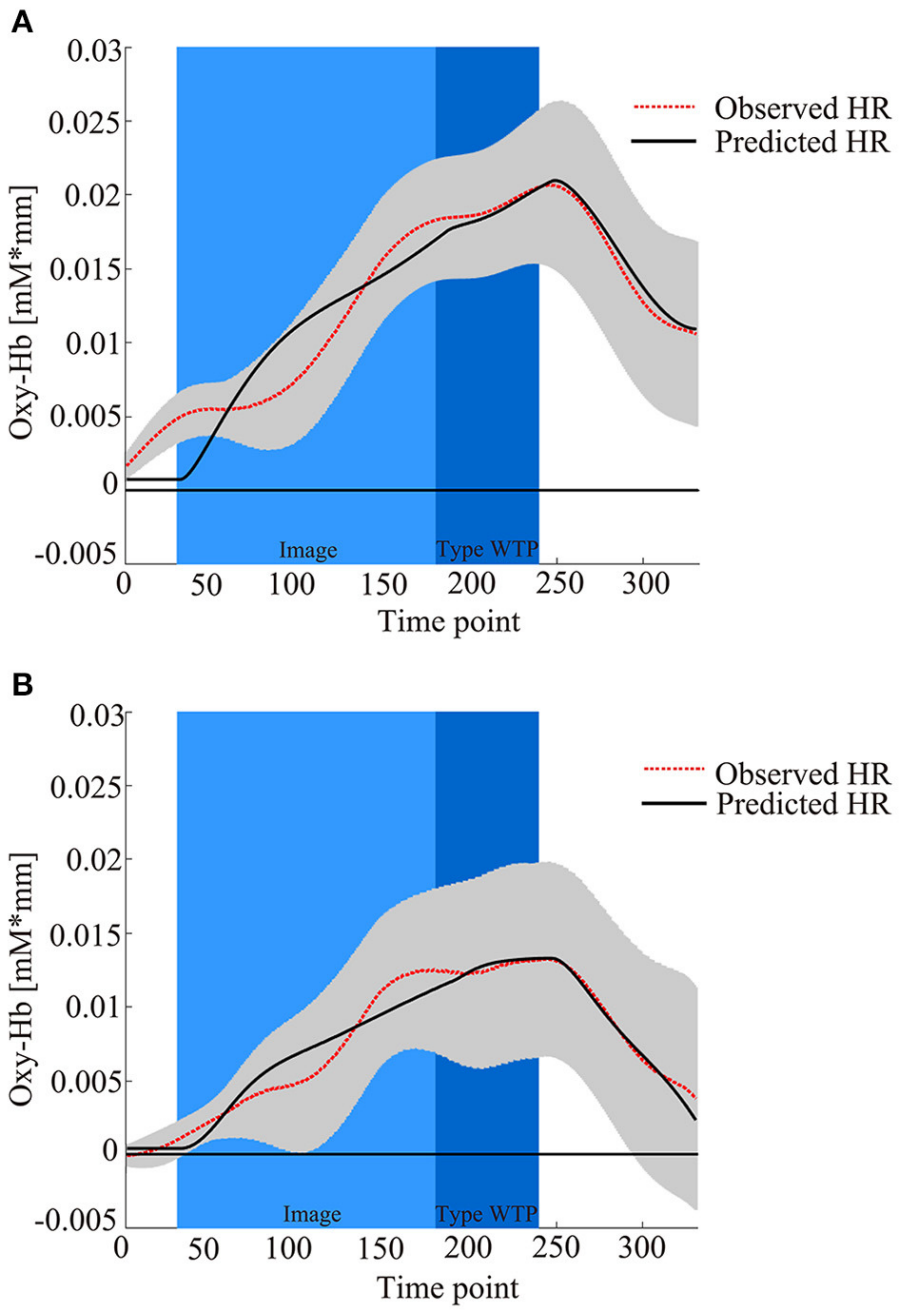
fNIRS time series data for all 52 channels during the trial procedure. The graphs show the observed and predicted time-series data for fNIRS from all channels for three products in **(A)** the DIY condition and **(B)** the Non-DIY condition, averaged across all participants. The light blue area represents product image presentation, the blue area represents WTP typing, the gray area represents standard deviation (SD), the dashed red line represents observed hemodynamic response, and the solid black line represents predicted hemodynamic response reflecting the composite contribution of all the regressors after personal adaptation.

Then, hemodynamic response data for the six products were blocked into two conditions (DIY, Non-DIY). Brain activation was obtained as β values by applying the value of τ_p_ calculated earlier to the blocked data (Plichta et al., 2006, 2007). Figure 4 shows the observed hemodynamic response and the predicted response, which was created by combining regressors adjusted for each participant with the personalized adaptive GLM. Thus, four β values for two conditions (DIY and Non-DIY) and two constants were calculated. β_1_ (DIY condition) and β_4_ (Non-DIY condition) were subjected to second-level analysis.

### Statistical analysis

For second-level analysis, we identified brain regions associated with the IKEA effect. Specifically, two β values for two conditions (DIY and Non-DIY) were subjected to second-level analysis. One-sample *t*-tests against zero were performed across participants for the null hypothesis that there is no change of hemodynamic response in a given channel. We used IBM SPSS statistical version 25 (IBM Inc., Armonk, NY, USA) and MATLAB2021 (The Mathworks Inc., Natick, MA, USA) to analyze behavioral and hemodynamic response data.

### Behavioral data for stimulus check

The WTP scores typed in the WTP evaluation task were subjected to a paired *t*-test for both conditions. This was to confirm that the IKEA effect occurred in all participants who subsequently underwent fNIRS data analysis.

### fNIRS data

In the second-level analysis-1, one-sample *t*-tests against zero (two tails) were conducted for both DIY and Non-DIY conditions in order to visualize the cortical activation under both conditions. In this analysis, we confirmed the putatively activated channels based on the effect sizes. It was necessary to consider a reasonable effect size for the data of 24 participants, to that end G^*^Power (release 3.1.9.7) was used (Faul et al., 2007). Power analysis was conducted under the conditions of sample size = 24 and one-sample *t*-test with α = 0.05 and power = 0.8 and a reasonable effect size of 0.60 was obtained based on the study by Cohen (1992). For this reason, we defined a reasonable effect size of 0.60 or greater as activation of brain function in this analysis. Traditionally, *p*-values are used as an index in functional brain analysis. However, *p*-values are an index for judging the rejection of the null hypothesis but not to judge the acceptance of the alternative hypothesis (Cohen, 1994). This is the first study to measure the IKEA effect in terms of cortical activation. Considering the exploratory nature of our study, we wanted to reduce beta (Type II errors) as much as possible. Therefore, we decided that it was justifiable to conduct the analysis using effect sizes in this study.

Subsequently, in the second-level analysis-2, we compared the cortical activation between DIY and Non-DIY conditions. The channels with an effect size of 0.60 or larger in the one-sample *t*-test in either of the two conditions were defined as Channels of Interest, and a paired *t*-test was conducted. As with second-level analysis-1, an effect size of 0.60 or larger was defined as activation.

## Results

### Behavioral analysis for stimulus check

**Figure 5:**
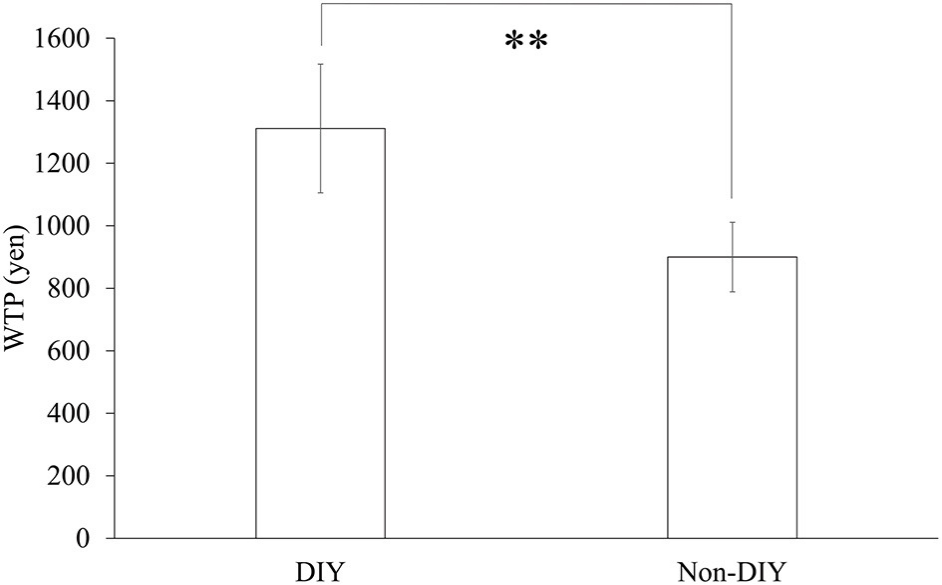
WTP scores for each condition. *N* = 24, error bars indicate *SE*, ^**^ indicates *p* < 0.01.

We performed a paired *t*-test on the WTP scores between both conditions. The results indicated significant differences between conditions as shown in Figure 5 [*t*_(23)_ = 3.018, *p* = 0.007, *d* = 0.64]. Thus, we confirmed that the IKEA effect occurred for 24 participants.

### fNIRS results

**Figure 6:**
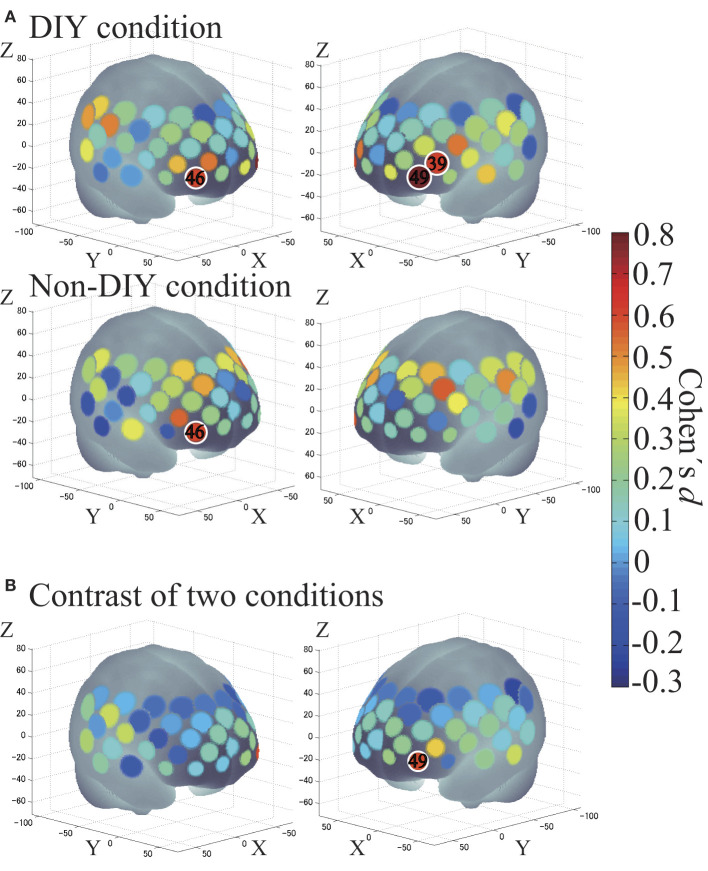
**(A)** Cortical activation for each condition. DIY condition on top, and Non-DIY condition on the bottom. **(B)** DIY minus Non-DIY contrast. Activated regions are shown according to the color bar. Areas circled in white represent an effect size of 0.6 or greater.

We performed one-sample *t*-tests against zero (two tails) to examine brain activation in each condition as second-level analysis-1 (Figure 6A). Channel 46 was activated in both DIY and Non-DIY conditions: *x* = 40, *y* = 64, *z* = −3 in MNI coordinates; R-IFG in LBPA40; and right FPC in BA [DIY condition: *t*_(23)_ = 3.11, *p* < 0.01, *d* = 0.65; Non-DIY condition: *t*_(23)_ = 2.94, *p* < 0.01, *d* = 0.61]. Channels activated only in the DIY condition were Channel 39: *x* = −48, *y* = 49, *z* = 10 in MNI coordinates; L-IFG in LBPA40; and L-DLPFC in BA [*t*_(23)_ = 2.95, *p* < 0.01, *d* = 0.62] and Channel 49: *x* = −38, *y* = 63, *z* = −2 in MNI coordinates; left middle frontal gyrus (L-MFG) in LBPA40; and left FPC in MRIcro [*t*_(23)_ = 3.77, *p* < 0.01, *d* = 0.79].

Subsequently, paired *t*-tests were performed to compare brain activation between the two conditions as second-level analysis-2 (Figure 6B). Channel 49 was activated more highly in the DIY condition than in the Non-DIY condition [*t*_(23)_ = 2.88, *p* < 0.01, *d* = 0.60].

## Discussion

### Overview

The purpose of this study was to examine the cognitive mechanisms of the IKEA effect. The results confirmed the activation of the R-IFG, L-IFG, and L-MFG in the DIY condition and of the R-IFG in the Non-DIY condition. These results suggest that two regions, the L-IFG and L-MFG, are associated with the IKEA effect. These areas correspond to the FPC and L-DLPFC, respectively, when classified by the Brodmann area. Below, we discuss the brain regions found activated in the current study and consider the possible cognitive mechanism underlying the IKEA effect.

### Cortical activations related to IKEA effect

First, the R-IFG activations in both conditions were relevant to the consideration of the WTP evaluation for all products. The R-IFG has been shown to be associated with economic decision-making in multiple studies using fMRI (Plassmann et al., 2007; Knutson et al., 2008; Christopoulos et al., 2009; Hare et al., 2010). In addition, the current results are in line with a previous fMRI study showing R-IFG activation associated with the endowment effect (Votinov et al., 2010). Many previous studies have mentioned that the right PFC is significantly activated for WTP measurements. However, in the present study, the right PFC activation for the IKEA effect, found in the DIY minus Non-DIY contrast, was significant while the activation for WTP was not. Therefore, it is possible that the left PFC activation might reflect a composite cognitive reaction for WTP in combination with the IKEA effect.

Next, possible roles of the L-IFG and L-MFG, which were activated only for DIY products, will be discussed. The L-IFG has been associated with past memory recall (Clark et al., 2015). In this experiment, the recall of DIY memories was expected to occur while participants were viewing pictures of the products during the WTP evaluation. In this sense, the L-IFG activation is relevant and may reflect the memory recall function occurring during the WTP evaluation of DIY products. The L-MFG is said to be related to encoding schema on memory retrieval (Webb et al., 2016). In this sense, it is reasonable to infer that the L-MFG was activated to possibly recall the DIY experience and encode it into monetary values.

Moreover, the activations in the FPC and in the L-DLPFC in the DIY condition were similar to those from previous studies measuring brain functions related to attachment (Yargiçoglu, 2022). Considering the report by Atakan (2011) describing that attachment occurs through DIY activities, these activations suggest that the brain regions related to attachment were recruited for the DIY condition, which probably evoked attachment to the DIY products assembled by each participant. Based these results, we suggest that the attachment to the products were generated during the DIY experience and the memory of the DIY experience was recalled by looking at pictures of the DIY products during the WTP measurement. On the other hand, Kikuchi et al. (2021) described a positive correlation between brain areas related to memory (PCC–anterior hippocampus connectivity) and evaluation of attachment and revealed that greater product attachment evoked increased connectivity in memory-related regions. Unfortunately, the current study could not examine activations of these areas because they are located beyond the reach of near-infrared light. Thus, we have to note that the current study presents only a partial view of the neural substrates underlying the IKEA effect that possibly involves an association between attachment and memory.

### Future perspectives and limitations

We must note that the IKEA effect is considered to be a compound effect that is a mixture of various cognitive biases. First, the endowment effect is considered to be similar to the IKEA effect (Kahneman et al., 1990). Norton et al. (2012), however, identified the difference between the two effects by recognizing that the IKEA effect leads to increased WTP scores more than the simple endowment effect does. Second, Shmueli et al. (2012) found that the IKEA effect increases product valuations depending on the difficulty of the assembly process. Third, Ling et al. (2020) showed that the IKEA effect is magnified by increasing consumer choice opportunities in product completion. Gibbs and Drolet (2003) found that consumers' energy levels increase the likelihood of selecting DIY products. In other words, the IKEA effect may increase with the participant's energy level. We believe that adjusting for these factors and conducting neuroimaging experiments may shed more light on the neural mechanism behind the IKEA effect.

Furthermore, the two factors, memory and attachment, which are considered important cognitive components of the IKEA effect, may also be related to other experiential consumption, such as the experience of eating. In the future, we believe that the present experimental design incorporating fNIRS can be used as a reference for examining neural substrates for other types of experiential consumption. To build on this, further research on quantifying the value of experiential consumption, taking advantage of the many strengths of fNIRS, is required to further investigate the mechanism of the IKEA effect. We believe that such research can be conducted from two promising perspectives. The first is to conduct research under conditions that more closely approximate daily life. The second is to conduct research using simultaneous measurements. In fact, in recent years, there has been increasing research on simultaneous measurement using fNIRS and other devices, as well as research on using fNIRS measurements under conditions similar to daily life (Pinti et al., 2015; Bhatt et al., 2019; Sargent et al., 2020). Building upon such research will make it possible to explore the mechanism of the IKEA effect and quantify the value of experiential consumption by taking advantage of these fNIRS strengths.

## Significance to neuroergonomics

In recent years, experiential consumption, which refers to purchases involving hedonic experiences, has been gathering attention in marketing research. Experiential consumption is closely related to cognitive biases, and among them, there is an interesting phenomenon called the IKEA effect, which is a cognitive bias in which a product is highly valued due to the experience of assembling it. Here we presented the first study, to our knowledge, that has explored the neural substrates of the IKEA effect using fNIRS and found that brain regions including the L-IFG and L-MFG are probably involved in the cognitive processing related to the IKEA effect. In particular, the L-MFG was more highly activated during the DIY condition than during the Non-DIY condition, and probably represents a core function of the IKEA effect. The current study has, for the first time, revealed the neural basis of the IKEA effect. In summary, this study suggests that the value of experiential consumption can be assessed using fNIRS-based neuroimaging and provides a novel approach to consumer neuroergonomics.

## Data availability statement

The raw data supporting the conclusions of this article will be made available by the authors, without undue reservation.

## Ethics statement

The studies involving human participants were reviewed and approved by Human Research Ethics Committee of Chuo University. The patients/participants provided their written informed consent to participate in this study.

## Author contributions

HO and TT contributed to the conception and design of the study. HO performed the experiments, analyzed data, and performed statistical analyses. YK and KN developed the statistical analysis plan. HO and ID wrote the manuscript. ID supervised this study. All authors have reviewed the manuscript and approved the final version for publication.
